# Association between sleep habits/disorders and emotional/behavioral problems among Japanese children

**DOI:** 10.1038/s41598-021-91050-4

**Published:** 2021-06-01

**Authors:** Masahiro Takeshima, Hidenobu Ohta, Tomoko Hosoya, Masakazu Okada, Yukako Iida, Aiko Moriwaki, Hidetoshi Takahashi, Yoko Kamio, Kazuo Mishima

**Affiliations:** 1 Department of Neuropsychiatry, Akita University Graduate School of Medicine, 1-1-1 Hondo, Akita City, Akita, 010-8543 Japan; 2 Graduate School of Integrated Frontier Science, Kyushu University, Fukuoka, Japan; 3 Faculty of Sport Science, Surugadai University, Saitama, Japan; 4 Department of Psychology, College of Education, Psychology and Human Studies, Aoyama Gakuin University, Tokyo, Japan; 5 Kochi Medical School Department of Child and Adolescent Psychiatry, Kochi University, Kochi, Japan; 6 Department of Preventive Intervention for Psychiatric Disorders, National Institute of Mental Health, National Center of Neurology and Psychiatry (NCNP), Tokyo, Japan; 7 Institute of Education and Human Development, Ochanomizu University, Tokyo, Japan; 8 Department of Sleep-Wake Disorders, National Institute of Mental Health, National Center of Neurology and Psychiatry (NCNP), Tokyo, Japan

**Keywords:** Sleep, Human behaviour

## Abstract

Actual sleep status and the association between sleep habits/disorders and emotional/behavioral problems among children in the development stage have not been fully clarified. A questionnaire survey was conducted on the sleep habits/disorders (Brief Child Sleep Questionnaire; BCSQ) and emotional/behavioral problems (Strengths and Difficulties Questionnaire; SDQ) of 87,548 children enrolled in ordinary classes in nine grade levels from the first grade of elementary school to the third grade of junior high school from December 2009 to April 2010. As school grade increased, children’s bedtimes were delayed and sleep duration was reduced by 2.0 h over the nine grade levels. Based on the BCSQ, 18.3% of children were judged to have some type of sleep disorder, and about 30% to 40% of children had sleep symptoms at bedtime, during sleep, and at wake time. Multiple regression analysis showed that emotional and behavioral problems were associated with presence of any sleep symptom, longer sleep latency, and longer awake time after sleep onset, whereas total sleep time was not. Sleep symptoms at wake time were most strongly associated with emotional and behavioral problems. Status of sleep habits/disorders should be considered when interpreting emotional/behavioral problems in school-age children.

## Introduction

Sleep plays an important role in child development. In addition to being associated with childhood body growth^1^, behavior^2^, and emotion^3^, sleep is also closely related to cognitive function^4^, school behavioral assessment^5,6^, and cautioning by teachers about behavior^7^. A recent systematic review of sleep habits (i.e., sleep onset time, wake time, and total sleep time [TST]) of children aged 0 to 12 years reported that children aged 6 to 12 years have an average sleep time of 9 h and 12 min, with the sleep time of children in Asian countries, including Japan, approximately 1 h shorter than that of children in other countries^8^.

Epidemiological studies in Europe and the United States suggest that approximately one-quarter of children have daily sleep problems such as lack of sleep or difficulty in waking, or sleep disorders such as insomnia, daytime hypersomnia, and sleep disordered breathing^9^. School-age children’s daytime cognitive function is known to be associated with not only sleep disorders^10^, but also low-quality sleep^11^. In fact, a longitudinal study following 1,492 children from the age of 5 months to 6 years reported that children who had regular short sleep duration (< 10 h) until 3.5 years of age had a 2.4- to 3.1-fold higher risk of low cognitive function at the age of 6 years than children who slept for 10 h or longer, regardless of their sleep duration in subsequent years^12^. In addition, numerous reports have indicated that sleep debt among children is associated not only with decreased arousal^13^, but also with hyperactivity^14^, inattention^15,16^, irritability^16^, poor mood^16^, and low threshold for frustration and distress^17^, which are often observed in children with attention deficit hyperactivity disorder (ADHD)^17^.

Sleep disorders in children have been recognized as an important early sign or risk factor for developing psychiatric disorders such as depression and developmental disorders^18^. Longitudinal studies have shown that persistent sleep problems during childhood (ages 5–9 years) predicted adulthood anxiety disorders (age 21 or 26 years)^19^. Retrospective studies on children have also reported that irregularities in sleep behavior are associated with subsequent mood disorders and the development of anxiety disorders^20^. The association with sleep problems has also been reported for children with autism spectrum disorder (ASD)^21–23^ and those with ADHD^23–25^.

While many studies have investigated the relationship between sleep problems, emotional/behavioral problems, and psychiatric disorders, in general, the extent and nature of sleep problems in children has not been fully clarified owing to insufficient sample sizes, leading to a lack of understanding in the fields of medical care, education, and support.

The purpose of this large-scale nationwide questionnaire study was to investigate actual sleep status, such as sleep habits/disorders, and its relationship with emotional/behavioral problems during childhood and mid-adolescence in a general population of children.

## Methods

### Subjects and data collection

**Figure 1:**
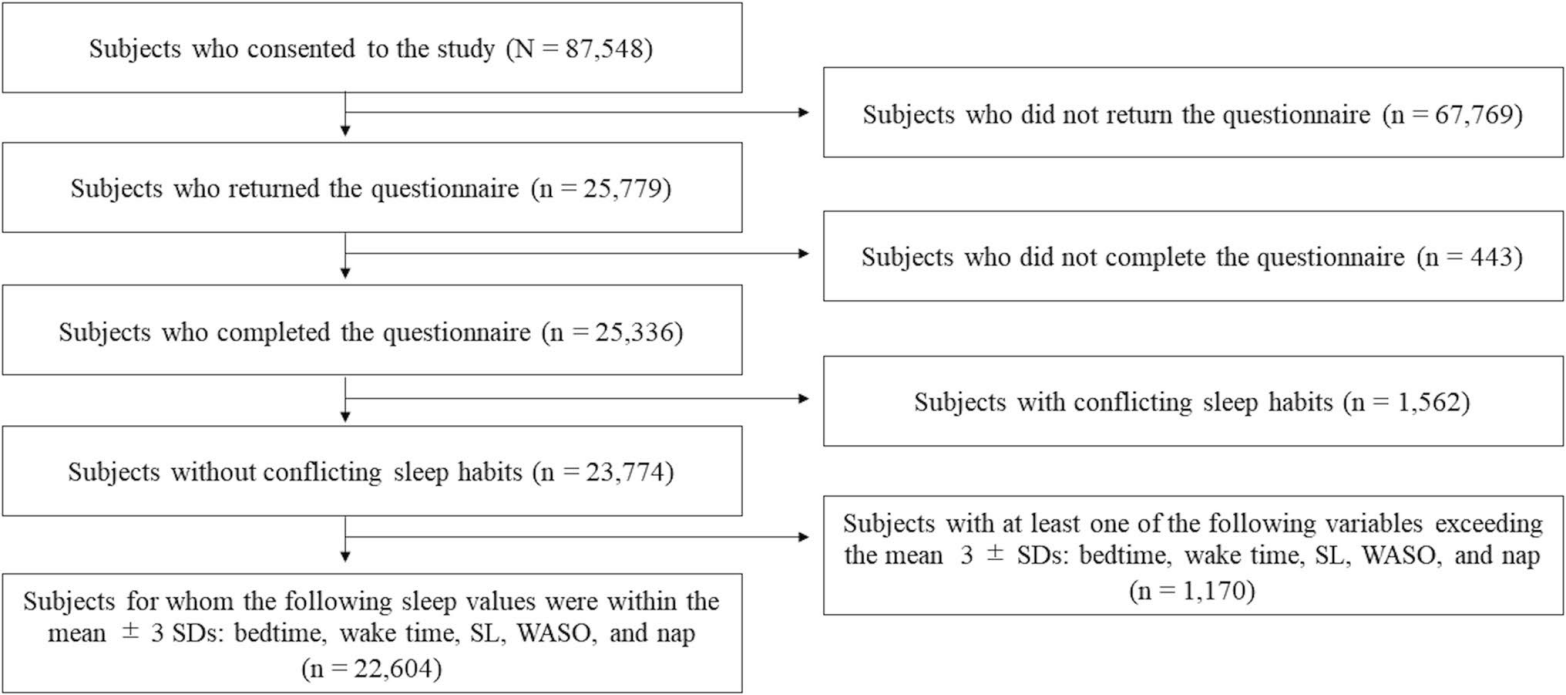
Selection of study subjects. Of the 87,548 subjects who were enrolled in the study, 25,779 responded to the questionnaire. Of the collected questionnaires, those of 443 subjects were excluded due to their responses having one or more deficiencies regarding indication of sex or age, or with the filling in of the questionnaire, 1,562 subjects were also excluded due to irrational sleep parameters (i.e., total sleep time is 0 h or less, time in bed plus nap is 24 h or more). Further, 1,170 subjects were excluded because sleep parameters habits (bedtime, wake time, sleep latency, wake after sleep onset, and nap) had outliers that exceeded the mean ± 3 standard deviations. Finally, the data of a total of 22,604 subjects were analyzed. Abbreviations: SD, standard deviation; SL, sleep latency; TST, total sleep time; WASO, wake after sleep onset.

The surveyed schools were selected nationwide with the assistance of the Ministry of Education, Culture, Sports, Science and Technology of Japan and the educational committees in each region. The subjects of the survey were 87,548 children (aged 6–15 years) enrolled in ordinary classes at 148 elementary schools and 71 junior high schools located in 10 regions from the north to south of Japan (Hokkaido, Akita, Saitama, Nagano, Toyama, Ishikawa, Fukui, Shiga, Tokushima, and Saga prefectures). Researchers and school officials explained the study to the parents, obtained their informed consent, and provided them the questionnaires. Parents completed the questionnaires and mailed them to a data entry company. Data received from December 2009 to the end of April 2010 were considered valid. In total, 25,779 questionnaires were collected (collection rate, 29.4%). Among the collected questionnaires, 443 subjects were excluded owing to deficient responses regarding sex or age, or problems with filling out the sleep questionnaire or the Strengths and Difficulties Questionnaire (SDQ). In addition, 1,562 subjects were excluded due to irrational sleep parameters (e.g., TST was 0 h or less, or time in bed [TIB] plus nap was 24 h or more). Another 1,170 subjects were excluded because certain sleep habits (bedtime, wake time, sleep latency [SL], wake after sleep onset [WASO], and nap) had outliers that exceeded the mean ± 3 standard deviations. Finally, data from a total of 22,604 subjects were analyzed for this study. Figure 1 shows the selection of subjects.

### Survey content

#### Sleep habits

Parents recorded sleep variables of the subject children, noting average bedtime, wake time, and nap duration over the course of 1 month. TST was calculated by subtracting sleep onset latency and WASO from time in bed (TIB), and sleep efficiency was calculated as the ratio of TST to TIB.

#### Brief child sleep questionnaire

The Brief Child Sleep Questionnaire (BCSQ) is a 19-item brief questionnaire that evaluates sleep disorders of children and adolescents over a period of a month. The Japanese version of the BCSQ has been confirmed for reliability and validity^26^. Three-way frequency assessments (1: rare [1 day/week or less]; 2: occasionally [2–4 days/week]; 3: almost always [5–7 days/week]) were used to evaluate four symptoms at bedtime (child falls asleep with rocking or rhythmic movements, child needs special object to fall asleep, etc.), nine symptoms during sleep (child talks during sleep, child is restless and moves a lot during sleep, etc.), five symptoms at wake time (child wakes up in a negative mood, child has difficulty getting out of bed in the morning, etc.), and one symptom during the day (child suddenly falls asleep in the middle of active behavior) (see Table S1). The presence of sleep symptoms (at bedtime, during sleep, at wake time, and during the day) was counted when one or more items were applicable. The cut-off value of the BCSQ was 24 points or more, and children with a total score equal to or greater than 24 points were considered to have some type of sleep disorder.

#### Strengths and difficulties questionnaire

The SDQ is a 25-item questionnaire that evaluates psychopathology and its severity in children and adolescents. Goodman developed the SDQ by adding additional items related to peer relationships and social competencies to the Rutter questionnaire^27,28^. The SDQ has been translated into more than 75 languages, including Japanese^29^. Questionnaires are divided into four difficulties subscales (emotional symptoms, behavioral conduct problems, hyperactivity/inattention, peer problems) and one subscale on prosocial behavior, which is a positive factor unlike the other four factors. Each item is evaluated on a 3-point scale (0: not applicable, 1: slightly applicable, 2: applicable), and each subscale is scored from 0 to 10. Total difficulties scores (TDS; 0 to 40 points) are calculated from four difficulties subscales (emotional symptoms, behavioral conduct problems, hyperactivity/inattention, and peer problems). In this study, we used the Japanese version of the SDQ^30^, which has been standardized (https://sdqinfo.org/norms/JapaneseNorms.html).

### Ethical procedures

This study was approved by the Ethics Committee of the National Center of Neurology and Psychiatry and adheres to the ethical guidelines of clinical and epidemiological research (No. XXXX-096 [20-8-2]).

### Statistical analysis

Statistical analyses were performed with SPSS Statistics 25.0 (IBM Corp. Armonk, NY). Summary measurements are presented as the mean ± standard deviation. The *t*-test was used to compare mean values, and the χ^2^ test was used to compare ratios of sex and other variables. Spearman correlation analysis was performed to investigate whether emotional and behavioral problems of children correlated with the following variables: age, sex, total BCSQ scores, sleep symptoms (at bedtime, during sleep, at wake time, and during the day) as measured by the BCSQ, and sleep habits (TST, TIB, SL, WASO, and nap). All variables with significant values (*P* < 0.3) in the Spearman correlation tests were included in the multiple regression analysis. To evaluate whether sleep disorders/habits were associated with emotional and behavioral problems, a multiple regression analysis was conducted with adjustment for other variables. Before running multiple regression analysis, multicollinearity was examined by calculating the variance inflation factor (VIF) and tolerance. The cut-off value was set at 3 for the VIF and 1 for tolerance^31^.

## Results

### Sleep habits reported by parents

Mean age was 10.3 ± 2.5 years. There were 11,537 boys (51.0%) and 11,067 girls (49.0%). The ratios of the subject population for each grade to the total subject population from the first grade of elementary school to the third grade of junior high school were as follows: 14.3%, 13.3%, 12.9%, 12.4%, 12.2%, 10.9%, 9.4%, 8.5%, and 6.0%. Table 1 shows changes in sleep habits by grade. In particular, bedtime changed greatly as school grade increased, and bedtime became progressively delayed from 21:06 in the first grade of elementary school to 23:18 in the third grade of junior high school. There was almost no change in wake time in students from elementary school to junior high school. Wake times of both elementary and junior high school students in all grades were in the range of 6:36–6:54. As the change in wake time was relatively small compared with the delay in bedtime, sleep duration at night (TIB, TST) decreased with increasing school grade, and shortened by 2.0 h over the nine grade levels from the first grade of elementary school to the third grade of junior high school.

**Table 1:** Sleep habits by school grade.

	Bedtime	Wake time	Nap (min)	SL (min)	WASO (min)	TIB (hour)	TST (hour)	SE (%)
Elementary school								
First grade	21:06 (36)	6:36 (24)	0.1 (2.1)	15.9 (10.9)	0.7 (2.5)	9.5 (0.6)	9.2 (0.6)	97.1 (2.0)
Second grade	21:17 (36)	6:36 (24)	0.1 (1.9)	16.0 (11.3)	0.6 (2.2)	9.4 (0.5)	9.1 (0.5)	97.1 (2.1)
Third grade	21:24 (36)	6:42 (0.4)	0.1 (2.0)	16.2 (11.7)	0.8 (3.0)	9.2 (0.6)	9.0 (0.6)	97.0 (2.3)
Fourth grade	21:36 (36)	6:42 (24)	0.2 (2.1)	16.3 (12.2)	0.8 (2.7)	9.0 (0.6)	8.7 (0.6)	96.8 (2.4)
Fifth grade	21:48 (36)	6:42 (24)	0.2 (2.5)	16.7 (13.0)	0.7 (2.9)	8.8 (0.6)	8.5 (0.6)	96.7 (2.6)
Sixth grade	22:06 (42)	6:42 (24)	0.2 (2.5)	16.5 (13.5)	0.8 (3.1)	8.6 (0.7)	8.3 (0.7)	96.7 (2.7)
Junior high school								
First grade	22:36 (42)	6:30 (30)	0.4 (3.6)	15.8 (13.8)	0.7 (3.3)	7.9 (0.7)	7.6 (0.7)	96.5 (3.1)
Second grade	22:54 (42)	6:36 (30)	0.6 (4.1)	15.9 (14.3)	0.8 (3.3)	7.7 (0.7)	7.4 (0.7)	96.4 (3.2)
Third grade	23:18 (42)	6:54 (30)	0.8 (5.1)	16.3 (14.5)	0.7 (3.5)	7.5 (0.7)	7.2 (0.7)	96.2 (3.3)
Overall	21:54 (54)	6:36 (24)	0.3 (2.8)	16.2 (12.6)	0.7 (2.9)	8.8 (0.9)	8.5 (0.9)	96.8 (2.6)

Continuous variables are expressed as the mean ± standard deviation.

*SE* sleep efficiency, *SL* sleep onset latency, *TIB* time in bed, *TST* total sleep time, *WASO* wake after sleep onset.

### Sleep disorders assessed by the BCSQ

Table 2 shows the percentage of patients judged to have suspected sleep disorders based on the BCSQ by grade. A total of 4,135 children (18.3%) were judged to have some type of sleep disorder; 34.5%, 32.9%, 41.7%, and 0.3% of these children experienced “twice a week or more” of at least one sleep-disorder item related to “at bedtime,” “during sleep,” “at wake time,” and “during the day,” respectively, indicating that most problems occurred at wake time. Moreover, sleep symptoms at bedtime and during sleep were more frequent in the lower grades. The proportion of sleep symptoms at wake time was lowest in sixth graders of elementary school. Table S2 shows the percentage of each sleep symptom that children experienced “twice a week or more” by grade.

**Table 2:** Brief child sleep questionnaire by school grade.

	Suspected sleep disorder	Symptoms at bedtime	Symptoms during sleep	Symptoms at wake time	Symptoms during the day
Elementary school					
First grade	24.8%	45.5%	41.9%	46.8%	0.4%
Second grade	21.5%	44.2%	38.3%	42.4%	0.2%
Third grade	19.3%	41.0%	35.7%	40.6%	0.3%
Fourth grade	19.7%	38.6%	34.5%	40.8%	0.2%
Fifth grade	17.8%	34.9%	32.7%	39.2%	0.3%
Sixth grade	15.3%	31.0%	30.6%	38.7%	0.2%
Junior high school					
First grade	14.0%	22.5%	25.7%	41.9%	0.5%
Second grade	12.9%	19.1%	23.4%	41.6%	0.4%
Third grade	11.2%	10.7%	19.1%	41.8%	0.1%
Overall	18.3%	34.5%	32.9%	41.7%	0.3%

### Emotional and behavioral problems of children assessed by the SDQ

Table 3 shows average scores for total scores and each subscale of the SDQ (emotional symptoms, conduct problems, hyperactivity/inattention, peer problems, and prosocial behavior) by school grade. Table 3 shows that scores decreased significantly as school grade year increased in several subscales, with the greatest decrease in hyperactivity/inattention (F[8, 22604] = 92.790; *P* < 0.001). There was a significant decrease in conduct problems (F[8, 22604] = 46.229; *P* < 0.001) and emotional symptoms (F[8, 22604] = 37.700; *P* < 0.001), whereas there was a relatively small decrease in prosocial behavior (F[8, 22604] = 9.903; *P* < 0.001). There was no significant difference in peer problems (F[8, 22604] = 1.924; *P* = 0.052).

**Table 3:** Strengths and difficulties questionnaire by school grade.

	TDS	Emotional symptoms	Conduct problems	Hyperactivity/inattention	Peer problems	Prosocial behavior
Elementary school						
First grade	8.7 (5.1)	1.7 (1.8)	2.1 (1.6)	3.4 (2.3)	1.5 (1.6)	6.2 (2.1)
Second grade	8.4 (5.0)	1.6 (1.8)	2.0 (1.6)	3.3 (2.2)	1.5 (1.5)	6.1 (2.1)
Third grade	7.9 (5.0)	1.5 (1.7)	1.9 (1.6)	3.0 (2.2)	1.5 (1.6)	6.2 (2.1)
Fourth grade	7.5 (4.9)	1.4 (1.7)	1.8 (1.5)	2.9 (2.2)	1.4 (1.5)	6.3 (2.1)
Fifth grade	7.2 (4.8)	1.3 (1.6)	1.7 (1.5)	2.7 (2.1)	1.5 (1.6)	6.2 (2.1)
Sixth grade	6.6 (4.8)	1.2 (1.6)	1.6 (1.4)	2.4 (2.0)	1.4 (1.6)	6.3 (2.1)
Junior high school						
First grade	6.9 (4.9)	1.2 (1.6)	1.6 (1.4)	2.6 (2.0)	1.4 (1.6)	5.9 (2.2)
Second grade	6.6 (4.8)	1.1 (1.6)	1.6 (1.4)	2.4 (2.0)	1.5 (1.6)	6.0 (2.2)
Third grade	6.2 (4.7)	1.1 (1.6)	1.5 (1.4)	2.2 (1.8)	1.5 (1.7)	5.9 (2.2)
Overall	7.5 (5.0)	1.4 (1.7)	1.8 (1.5)	2.9 (2.2)	1.5 (1.6)	6.2 (2.1)

Continuous variables are expressed as the mean ± standard deviation.

*TDS* total difficulty score.

### Association between sleep habits/disorders and emotional and behavioral problems of children

#### Correlation analysis

Spearman correlation analysis demonstrated that all variables other than nap significantly correlated with emotional and behavioral problems of children (see Table S3). The strength of the relationship ranged from none to moderate^32^: the correlation coefficient was 0.379 for total BCSQ scores, 0.277 for symptoms at wake time, 0.260 for symptoms at bedtime, 0.212 for symptoms during sleep, − 0.181 for age, 0.133 for WASO, 0.131 for TIB, 0.103 for SL, 0.099 for TST, 0.039 for symptoms during the day, and − 0.09 for male sex.

#### Multiple regression analysis

All variables other than TIB were under cut-off values for VIF and tolerance. However, TIB exceeded the cut-off values: 3.56 for VIF and 2.773 for tolerance. Therefore, TIB was not included in the multiple regression analysis. Table 4 shows results of the multiple regression analysis for emotional and behavioral problems. Analysis of TDS in Model 1, which was adjusted for age, sex, and total BCSQ scores, found a standardized partial regression coefficient of − 0.104 for age (*P* < 0.001), − 0.102 for sex (*P* < 0.001), and 0.385 for total BCSQ scores (*P* < 0.001). In Model 2, scores of four BCSQ subcategories were added to Model 1. The four BCSQ subcategories consist of symptoms at bedtime, during sleep, at wake time, and during the day. Analysis of Model 2 demonstrates that the standardized partial regression coefficient for TDS was − 0.102 for age (*P* < 0.001), − 0.098 for sex (*P* < 0.001), 0.161 for symptoms at bedtime (*P* < 0.001), 0.121 for symptoms during sleep (*P* < 0.001), 0.212 for symptoms at wake time (*P* < 0.001), and 0.035 for symptoms during the day (*P* < 0.001). In Model 3, which adds sleep habits such as TST, SL, and WASO to Model 2, the standardized partial regression coefficient for TDS was − 0.100 for age (*P* < 0.001), − 0.099 for sex (*P* < 0.001), 0.150 for symptoms at bedtime (*P* < 0.001), 0.115 for symptoms during sleep (*P* < 0.001), 0.207 for symptoms at wake time (*P* < 0.001), 0.034 for symptoms during the day (*P* < 0.001), 0.009 for TST (*P* = 0.308), 0.076 for SL (*P* < 0.001), and 0.078 for WASO (*P* < 0.001), indicating that younger age, male sex, presence of any sleep symptoms, longer SL, and longer WASO were associated with TDS, whereas TST was not.

**Table 4:** Multiple regression analysis of sleep problems (sleep disorders and sleep habits) on TDS (emotional and behavioral problems).

	Standardized partial regression coefficient		
	Model 1	Model 2	Model 3
Age	− 0.104**	− 0.102**	− 0.100**
Sex	− 0.102**	− 0.098**	− 0.099**
BCSQ total score	0.385**		
Symptoms at bedtime		0.161**	0.150**
Symptoms during sleep		0.121**	0.115**
Symptoms at wake time		0.212**	0.207**
Symptoms during the day		0.035**	0.034**
TST			0.009
SL			0.076**
WASO			0.078**
Adjusted R^2^	0.179**	0.153**	0.166**

BCSQ total score consists of the four subcategories of symptoms at bedtime, during sleep, at wake time, and during the day.

**P < 0.01, *P < 0.05.

*SL* sleep latency, *TST* total sleep time, *WASO* wake after sleep onset.

## Discussion

In an analysis of the BCSQ and SDQ, this study found three significant findings concerning the sleep and emotional properties of Japanese children in elementary and junior high schools. First, our study indicates that Japanese school-age children do not get enough sleep. Mean sleep duration was 8.5 h (6–12 years: 8.3–9.2 h; 13–15 years: 7.2–7.6 h), with sleep duration decreasing as grade increased. Sleep duration recommended by the National Sleep Foundation was 9 to 11 h for elementary school children (aged 6–13) and 8 to 10 h for adolescents (aged 14–17)^33^, suggesting that many children and adolescents do not meet recommended sleep duration in either age group. Previous studies have shown that in childhood, decreased sleep duration in daily life is mainly caused by delayed bedtime, which becomes progressively delayed with age^33–37^. The present study also found that bedtime became remarkably delayed as school grade increased, becoming 2.2 h (16.5 min per year) later over the nine grade levels between the first grade of elementary school and the third grade of junior high school. In contrast, wake time, which is greatly influenced by social factors related to school attendance, remained almost unchanged during the same period. As a result, total sleep duration was reduced by 2.0 h (15.0 min per year of age), which is consistent with previous studies^38^. The present study also demonstrated that the greatest change over a previous year was seen in the first year of junior high school, which showed a bedtime that was 30 min later and a 49-min overall reduction in sleep duration. This is likely due to the large change in lifestyle that children in that school year experience, including an increased study load and early morning scheduling of extracurricular school activities such as sports club activities. As such, careful attention should be paid to changes in sleep habits of junior high school students. In addition, the dramatic spread of smartphones and games among children over the past decade after the investigation might further change the lifestyle of children, which could secondarily cause sleep, emotional, and behavioral problems^39^. Therefore, further research is warranted to examine the recent sleep and mental health of children.

Second, by using the BCSQ, our study demonstrated that symptoms of suspected sleep disorders at wake time tended to decrease with age among elementary school students but increased with age among junior high school students. In particular, complaints of sleepiness during the day increased in junior high school students compared with elementary school students, indicating that sufficient sleep duration was less likely to occur in junior high school students (Table 2). Moreover, symptoms at both bedtime and wake time were frequently observed among children at the lower grades of elementary school, suggesting that a sizable number of them had insufficient sleep and physiologically inappropriate bedtimes and wake times. The greatest determinant of an individual’s sleep habits is thought to be their chronotype (an individual’s morning-night orientation)^40^, which is reported to be established by the first half of school life^41^. When children need to adopt an earlier sleep phase to suit changes in their social life, such as adapting to early school hours, individuals with a strong night chronotype have difficulty advancing their sleep onset time, resulting in accumulated sleep debt^42,43^. The present study suggests that careful setting of the sleep–wake schedule based on an individual’s biological clock is required, especially for lower-grade elementary school children whose daily schedule is greatly changed owing to school enrollment. The BCSQ also indicated that the percentage of children suspected of having sleep disorders was 18.3% (11.2% to 24.8%), which is lower than the 66.2% to 76.1% reported in previous studies using the Children’s Sleep Habits Questionnaire (CSHQ)^26,44,45^. Sleep problems reported in previous studies using the CSHQ include sleep–wake disorders as well as sleep habit problems such as night-owl lifestyle and sleep debt^26,44,45^. On the other hand, the BCSQ is a screening scale of the main symptoms of sleep–wake disorders often seen in children^26^, and the BCSQ might extract children with more severe sleep problems compared with the CSHQ.

Third, this study suggests that emotional and behavioral problems in children can be associated with sleep disorders even when adjusting for covariates such as age, sex, and sleep habits. In Model 1, multiple regression analysis demonstrated that children with suspected sleep disorders assessed by the BCSQ tended to have more emotional and behavioral problems as assessed by the SDQ, indicating a negative association of sleep disorders with emotional and behavioral problems in children. Actually, the pathophysiological linkage between neurodevelopmental disorders and sleep disorders has not been fully elucidated, although children with ADHD and ASD are reported to have higher comorbidity for sleep disorders such as insomnia, circadian sleep–wake disorders, sleep-related respiratory disorders, restless leg syndrome, and periodic limb movement disorders^46–48^.

In Model 2, which includes the four BCSQ subcategories rather than the total BCSQ score, analysis showed that sleep symptoms observed in all subcategories (“at bedtime,” “during sleep,” “at wake,” and “during the day”) might be associated with child emotional and behavioral problems. In particular, the sleep symptom “at wake time” might have a more significant negative association with child mental health than the other three subcategories.

In Model 3, which adds parent-reported sleep habits to Model 2, analysis indicates that longer SL and more WASO might be negatively associated with emotional and behavioral problems, whereas TST might not be significantly associated with emotional and behavioral problems in children. Many sleep–wake disorders are commonly accompanied by insomnia symptoms, such as difficulty falling asleep or nocturnal awakenings^49^. In fact, insomnia symptoms have been reported to be associated with emotional and behavioral problems^50^. In this study, prolonged SL and WASO were also significantly associated with emotional and behavioral problems. On the other hand, although some previous studies have shown that short sleep duration is associated with emotional and behavioral problems, in this study, TST was not associated with emotional and behavioral problems^51–53^. The reasons for the discrepancy between the results of this study and previous studies are unclear. Possible reasons are that (1) the sensitivity of TST, which is calculated indirectly by subtracting SL and WASO from TIB, may have been low compared with the abovementioned insomnia symptoms such as difficulty falling asleep and nocturnal awakenings, which are easily perceived by parents who are observers of the subject children’s sleep state; (2) optimal sleep duration varies from person to person^54^; and (3) the subjects’ ages in this study were wide-ranging from 6 to 15 years old.

A few limitations should be considered when interpreting the results. The first limitation of this study is the low collection rate. Second, data collected through the children’s parents might be inaccurate because questionnaire responses were greatly influenced by subjective parental evaluations. Third, we used the BCSQ with children aged 6–15 years, but the BCSQ has not been validated in children aged ≥ 13 years^26^.

The clinical implications of this study, which used a standardized children’s emotional and behavioral problems and sleep problems questionnaire, showed that sleep symptoms are associated with emotional and behavioral problems in a large population of Japanese children. The present findings suggest that sleep disorders should be considered when dealing with children with emotional and behavioral problems and that appropriate therapeutic interventions should be provided as needed. Future research will hopefully adopt more sophisticated designs, including assessment of objective sleep symptoms using actigraphy and other methods.

In conclusion, a survey of a large population of Japanese children using a standardized questionnaire showed that (1) Japanese children do not get enough sleep; (2) as a result, many children had symptoms at wake time that could be attributed to their sleep debt; and (3) independent of sleep habits, symptoms at bedtime, during sleep, at wake time, and during the day are associated with emotional and behavioral problems in children. Because this is a cross-sectional study, it remains unresolved whether children’s sleep symptoms are precursors to, exacerbators of, or comorbidities with pathological linkages of emotional and behavioral problems. If future cohort and clinical trial research provide insights that suggest that appropriate therapeutic interventions for sleep disorders might help children with emotional and behavioral problems, it would be very informative for child and adolescent psychiatry.

## Supplementary Information

Supplementary Information 1.

## Data Availability

The datasets used and analyzed during the current study are available from the corresponding author on reasonable request.
