# Supplementary material for: Association between sleep habits/disorders and emotional/behavioral problems among Japanese children
Source: Sci Rep. 2021 Jun 1;11:11438. doi: 10.1038/s41598-021-91050-4 (PMC8169700; doi:10.1038/s41598-021-91050-4)
Supplement: Supplementary file 1 — Supplementary Information 1. [file 41598_2021_91050_MOESM1_ESM.pdf]

Supplementary material for

Association between sleep habits/disorders and emotional/behavioral problems among Japanese children

Masahiro Takeshima, M.D, Ph.D.[1], Hidenobu Ohta, M.D, Ph.D.[1], Tomoko Hosoya, M.D.[1], Masakazu Okada, Ph.D.[2], Yukako Iida, Ph.D.[3], Aiko Moriwaki, Ph.D.[4], Hidetoshi Takahashi, M.D, Ph.D.[5], Yoko Kamio, M.D, Ph.D.[6,7], Kazuo Mishima, M.D, Ph.D.[1,8]

[1]Department of Neuropsychiatry, Akita University Graduate School of Medicine, Akita, Japan

[2] Graduate School of Integrated Frontier Science, Kyushu University, Fukuoka, Japan

[3] Faculty of Sport Science, Surugadai University, Saitama, Japan

[4] Department of Psychology, College of Education, Psychology and Human Studies, Aoyama gakuin University, Tokyo, Japan

[5] Kochi Medical School Department of Child and Adolescent Psychiatry, Kochi University, Kochi, Japan

[6] Department of Preventive Intervention for Psychiatric Disorders, National Institute of Mental Health, National Center of Neurology and Psychiatry (NCNP), Tokyo, Japan

[7] Institute of Education and Human Development, Ochanomizu University, Tokyo, Japan

[8] Department of Sleep-Wake Disorders, National Institute of Mental Health, National Center of Neurology and Psychiatry (NCNP), Tokyo, Japan

Contents

Table S1. Brief Child Sleep Questionnaire (BCSQ).

Table S2. Sleep symptoms experienced twice or more a week by grade level.

Table S3. Associations between emotional and behavioral problems with sleep problems (sleep habits and sleep disorders) evaluated by Spearman correlations.

Table S1. Brief Child Sleep Questionnaire (BCSQ).

Symptoms at bedtime	
1.	Child falls asleep with rocking or rhythmic movements
2.	Child needs special object to fall asleep (doll, special blanket, etc.)
3.	Child resists going to bed at bedtime
4.	Child is afraid of sleeping in the dark
Symptoms during sleep	
5.	Child talks during sleep
6.	Child is restless and moves a lot during sleep
7.	Child sleepwalks during the night
8.	Child grinds teeth during sleep (your dentist may have told you this)
9.	Child snores loudly
10.	Child seems to stop breathing during sleep
11.	Child snorts and/or gasps during sleep
12.	Child awakens during night screaming, sweating, and inconsolable
13.	Child awakens alarmed by a frightening dream
Symptoms at wake time	
14.	Child wakes up in negative mood
15.	Child has difficulty getting out of bed in the morning
16.	Child takes a long time to become alert in the morning
17.	Child wakes up very early in the morning
18.	Child has a good appetite in the morning
Symptoms during the day	

19.	Child suddenly falls asleep in the middle of active behavior

Table S2. Sleep symptoms experienced twice or more a week by grade level.

	BCSQ 1	BCSQ 2	BCSQ 3	BCSQ 4	BCSQ 5
Elementary School					
First grade	1.9%	20.9%	19.8%	20.5%	17.9%
Second grade	1.7%	19.1%	19.9%	20.4%	18.3%
Third grade	1.4%	16.1%	19.7%	17.9%	17.6%
Fourth grade	1.4%	14.5%	18.9%	17.1%	18.5%
Fifth grade	1.1%	12.0%	18.9%	13.2%	15.4%
Sixth grade	1.1%	8.9%	18.2%	10.5%	15.8%
Junior High School					
First grade	1.0%	6.4%	13.1%	6.2%	13.5%
Second grade	1.1%	5.8%	10.8%	5.0%	11.5%
Third grade	0.2%	3.3%	6.1%	1.9%	8.9%
Overall	1.3%	13.2%	17.2%	14.0%	16.0%

	BCSQ 6	BCSQ 7	BCSQ 8	BCSQ 9	BCSQ 10
Elementary School					
First grade	10.6%	0.7%	18.9%	10.3%	1.9%
Second grade	9.5%	0.9%	15.6%	9.1%	2.1%
Third grade	9.1%	1.0%	13.4%	9.4%	1.2%
Fourth grade	9.4%	1.1%	12.5%	8.3%	1.2%
Fifth grade	8.2%	0.7%	12.7%	9.4%	1.6%
Sixth grade	7.1%	1.1%	10.2%	8.4%	0.7%
Junior High School					

First grade	4.4%	0.2%	8.6%	7.9%	0.9%
Second grade	3.4%	0.1%	9.1%	7.5%	0.6%
Third grade	2.6%	0.4%	6.3%	5.0%	0.7%
Overall	7.8%	0.7%	12.7%	8.7%	1.3%

	BCSQ 11	BCSQ 12	BCSQ 13	BCSQ 14	BCSQ 15
Elementary School					
First grade	1.9%	0.7%	2.1%	23.9%	33.9%
Second grade	1.5%	0.8%	2.1%	20.0%	30.7%
Third grade	1.2%	0.3%	1.5%	17.6%	29.3%
Fourth grade	0.7%	0.6%	1.9%	18.7%	30.1%
Fifth grade	1.0%	0.5%	1.9%	16.1%	31.1%
Sixth grade	0.6%	0.4%	1.1%	16.8%	30.5%
Junior High School					
First grade	0.7%	0.4%	1.1%	18.2%	31.8%
Second grade	0.6%	0.1%	0.8%	19.1%	32.7%
Third grade	0.2%	0.1%	0.7%	19.5%	34.3%
Overall	1.1%	0.5%	1.6%	19.0%	31.4%

	BCSQ 16	BCSQ 17	BCSQ 18	BCSQ 19	
Elementary School					
First grade	21.9%	2.3%	12.7%	0.4%	
Second grade	19.4%	2.0%	10.0%	0.2%	
Third grade	18.3%	2.6%	8.5%	0.3%	
Fourth grade	18.6%	1.7%	7.8%	0.2%	

Fifth grade	17.8%	1.6%	7.5%	0.3%
Sixth grade	18.0%	1.3%	7.0%	0.2%
Junior High School				
First grade	20.1%	1.3%	7.2%	0.5%
Second grade	19.0%	0.9%	6.3%	0.4%
Third grade	21.1%	0.6%	7.8%	0.1%
Overall	19.3%	1.7%	8.6%	0.3%

Abbreviations: BCSQ, Brief Child Sleep Questionnaire; BCSQ 1, child falls asleep with rocking or rhythmic movements; BCSQ 2, child needs special object to fall asleep (doll, special blanket, etc.); BCSQ 3, child resists going to bed at bedtime; BCSQ 4, child is afraid of sleeping in the dark; BCSQ 5, child talks during sleep; BCSQ 6, child is restless and moves a lot during sleep; BCSQ 7, child sleepwalks during the night; BCSQ 8, child grinds teeth during sleep (your dentist may have told you this); BCSQ 9, child snores loudly; BCSQ 10, child seems to stop breathing during sleep; BCSQ 11, child snorts and/or gasps during sleep; BCSQ 12, child awakens during night screaming, sweating, and inconsolable; BCSQ 13, child awakens alarmed by a frightening dream; BCSQ 14, child wakes up in negative mood; BCSQ 15, child has difficulty getting out of bed in the morning; BCSQ 16, child takes a long time to become alert in the morning; BCSQ 17, child wakes up very early in the morning; BCSQ 18, child has a good appetite in the morning; BCSQ 19, child suddenly falls asleep in the middle of active behavior.

Table S3. Associations between emotional and behavioral problems with sleep problems (sleep habits and sleep disorders) evaluated by Spearman correlations.

Variables	R^2	*p*-value
Demographic data		
Age	0.033	0.000
Sex	0.008	0.000
BCSQ		
Total BCSQ score	0.144	0.000
Symptoms at wake time	0.077	0.000
Symptoms at bedtime	0.068	0.000
Symptoms during sleep	0.045	0.000
Symptoms during the day	0.002	0.000
Sleep habits		
WASO	0.018	0.000
TIB	0.017	0.000
Bedtime	0.012	0.000
SL	0.011	0.000
SE	0.010	0.000
TST	0.010	0.000
Wake time	0.002	0.000
Nap	0.000	0.527

P values with significant results are labeled with an asterisk.

Abbreviations: BCSQ, Brief Child Sleep Questionnaire; SE, sleep efficiency; SL, sleep latency; TIB, time in bed; TST, total sleep time; WASO, wake after sleep onset.
